# Fractionating autism based on neuroanatomical normative modeling

**DOI:** 10.1038/s41398-020-01057-0

**Published:** 2020-11-06

**Authors:** Mariam Zabihi, Dorothea L. Floris, Seyed Mostafa Kia, Thomas Wolfers, Julian Tillmann, Alberto Llera Arenas, Carolin Moessnang, Tobias Banaschewski, Rosemary Holt, Simon Baron-Cohen, Eva Loth, Tony Charman, Thomas Bourgeron, Declan Murphy, Christine Ecker, Jan K. Buitelaar, Christian F. Beckmann, Andre Marquand, The EU-AIMS LEAP Group

**Affiliations:** 1 Donders Institute for Brain, Cognition and Behavior, Radboud University Nijmegen, Nijmegen, The Netherlands; 2 Department for Cognitive Neuroscience, Radboud University Medical Center Nijmegen, Nijmegen, The Netherlands; 3 Division of Mental Health and Addiction, Norwegian Centre for Mental Disorders Research (NORMENT), University of Oslo & Oslo University Hospital, Oslo, Norway; 4 Department of Psychology, University of Oslo, Oslo, Norway; 5 Department of Psychology, Institute of Psychiatry, Psychology, & Neuroscience, King’s College London, London, UK; 6 Department of Applied Psychology: Health, Development, Enhancement, and Intervention, University of Vienna, Vienna, Austria; 7 Department of Psychiatry and Psychotherapy, Central Institute of Mental Health, University of Heidelberg, Mannheim, Germany; 8 Department of Child and Adolescent Psychiatry and Psychotherapy, Central Institute of Mental Health, Medical Faculty Mannheim/Heidelberg University, Mannheim, Germany; 9 Autism Research Centre, Department of Psychiatry, University of Cambridge, Cambridge, UK; 10 Sackler Institute for Translational Neurodevelopment, Institute of Psychiatry, Psychology & Neuroscience, King’s College London, London, UK; 11 Department of Forensic and Neurodevelopmental Sciences, Institute of Psychiatry, Psychology & Neuroscience, King’s College London, London, UK; 12 Human Genetics and Cognitive Functions, Institut Pasteur, UMR3571 CNRS, Université de Paris, Paris, France; 13 Department of Child and Adolescent Psychiatry, Psychosomatics and Psychotherapy, University Hospital Frankfurt am Main, Goethe University, Frankfurt, Germany; 14 Karakter Child and Adolescent Psychiatry University Centre, Nijmegen, The Netherlands; 15 Centre for Functional MRI of the Brain, University of Oxford, Oxford, UK; 16 Department of Neuroimaging, Institute of Psychiatry, Psychology, & Neuroscience, King’s College London, London, UK

**Keywords:** Predictive markers, Psychology, Autism spectrum disorders

## Abstract

Autism is a complex neurodevelopmental condition with substantial phenotypic, biological, and etiologic heterogeneity. It remains a challenge to identify biomarkers to stratify autism into replicable cognitive or biological subtypes. Here, we aim to introduce a novel methodological framework for parsing neuroanatomical subtypes within a large cohort of individuals with autism. We used cortical thickness (CT) in a large and well-characterized sample of 316 participants with autism (88 female, age mean: 17.2 ± 5.7) and 206 with neurotypical development (79 female, age mean: 17.5 ± 6.1) aged 6–31 years across six sites from the EU-AIMS multi-center Longitudinal European Autism Project. Five biologically based putative subtypes were derived using normative modeling of CT and spectral clustering. Three of these clusters showed relatively widespread decreased CT and two showed relatively increased CT. These subtypes showed morphometric differences from one another, providing a potential explanation for inconsistent case–control findings in autism, and loaded differentially and more strongly onto symptoms and polygenic risk, indicating a dilution of clinical effects across heterogeneous cohorts. Our results provide an important step towards parsing the heterogeneous neurobiology of autism.

## Introduction

Autism is a neurodevelopmental condition marked by impairments in social communication and interaction, alongside restricted and repetitive behaviors and sensory atypicalities^1^. It is still diagnosed based on behavioral observations and no validated biomarkers are available which could support diagnosis, stratification, or clinical management^2^. Efforts to identify replicable cognitive and biological substrates of the condition have been hampered by the pronounced biological, developmental, and clinical heterogeneity of individuals with autism^3–7^. Standard case–control analytical approaches ignore such heterogeneity and assume homogeneous diagnostic entities^8^. Therefore, case–control findings have been highly inconsistent for most neuroimaging derived measures^9–13^, for instance, with some studies reporting increases in cortical thickness (CT)^14,15^, others decreases^16^ and others no significant findings, even within the same brain regions. Moreover, the size of reported case–control effects has decreased over time as diagnostic procedures have become more inclusive thereby sampling a broader range of the clinical phenotype^17,18^, causing some to question the notion of biological heterogeneity in autism^18^. Case–control approaches are useful when there are consistent and well-defined alterations in the clinical population. However, the case–control paradigm cannot by definition unravel autism-related heterogeneity in the absence of such consistent alterations. In other words, inconclusive neuroimaging findings are likely the result of such case–control comparisons diluting consistent alterations within more biologically homogenous subgroups of individuals with autism. Thus, the identification of meaningful subtypes within autism is of utmost importance to facilitate biomarker discovery.

Despite considerable efforts in identifying biologically significant subtypes in autism^19–28^, no consensus has been reached, again due to the high phenotypic and neurobiological variation in autism. Most approaches to stratifying autism have employed clustering algorithms for symptomatology^29–38^. However, there is no guarantee that such behaviorally-defined clusters will map cleanly onto distinct biological mechanisms or outcomes. Moreover, the biologically-defined clustering approaches mostly follow the description of the condition on the group level and recapitulate the case–control paradigm, which often fails to fully capture the complexity of inter-individual alterations^5,27,39^. Thus, the first step towards subtyping autism is to chart the heterogeneity by moving away from the “average patient”, permitting inferences at the individual level. Normative modeling is one data-driven approach that provides a means to achieve this. We have previously applied normative modeling to autism to map neuroanatomical variability at the level of the individual^40^. This analysis showed highly individualized patterns of atypicality across nearly the entire cortex in different individuals. However, in order to better understand the nature of the underlying alterations and to move toward clinically useful stratifications of individuals with autism, it is necessary to go further. Specifically, it is necessary to summarize these complex and highly individualized patterns of deviation into a small number of interpretable neurobiological subtypes. To do so, we build on our previous work that used normative modeling^40^ further to find subtypes within the cohort. Here, we achieve this objective by combining normative modeling with clustering, which is appealing for three reasons: first, since the normative range is defined with respect to a supervised mapping between biology and covariates relevant to the disorder (while also accounting for nuisance variation), this allows the clustering algorithm to focus on clinically relevant variation. In contrast, clustering algorithms that operate in a purely unsupervised manner will likely just detect nuisance variation, which is usually of a larger magnitude than clinically relevant effects. Second, the normative model rescales different variables to a common (normative) reference range, yielding clusters that are more interpretable since they are defined with respect to a neurotypical reference group. Third, since the normative model can be learned on very large samples, this allows us to capitalize on big data cohorts to better capture the heterogeneity within clinical cohorts.

We apply this approach to a large and comprehensively characterized autism cohort recruited as part of the EU-AIMS Longitudinal European Autism Project (LEAP)^41^, which captures a broad range of the autism phenotype, along with extensive clinical phenotyping, multiple neurobiological assessments, and genetics. While normative modeling can predict various brain measures, here we focused on cortical thickness (CT) because firstly, alterations in CT have been extensively reported in different autism studies and secondly, it is a reliable measure of cortical morphology in autism^14,16,42–47^. Similar to other applications in neuroimaging^48–50^, we extensively validate the derived clusters. Specifically, we evaluate their stability, out-of-sample prediction capability, and test for associations with clinical, demographic, and genetic measures. We show that with the right analytical approaches, biological heterogeneity is quantifiable and can be used to derive a stratification of individuals where heterogeneity in the clinical presentation is reduced.

## Methods

We included 206 neurotypical (NT) (79 female, aged 17.5 ± 6.1 years) and 316 participants with autism (88 female, aged 17.2 ± 5.7) including mild intellectual disability participants across 6 sites from the EU-AIMS LEAP sample^41^. All participants were scanned with a T1-weighted imaging protocol, and FreeSurfer (v5.3)^51^ was used to estimate measures of regional CT. This sample has been described in detail previously^41^ as have the normative models that form the basis for this work^40^. However, we briefly summarize the clinical recruitment procedures, sample characteristics, processing steps, and procedures for normative modeling in the Supplementary Methods and Supplementary Table S1.

A methodological overview of our approach is shown in Supplementary Fig. S1 and full details are provided in the Supplementary Methods. Briefly, for each participant, we estimated normative models to predict vertex-wise cortical thickness as a function of age and sex plus nuisance covariates including full-scale IQ, plus dummy coded site variables and the Freesurfer Euler number (a proxy for scan quality) using Gaussian process regression. Then, we estimated normative probability maps (NPMs), which quantify the deviation from normative CT, by subtracting the predicted from true CT, divided by the estimated variance at each vertex to construct a subject- and vertex-specific *Z*-score.

In order to partition the autism cohort into sub-clusters on the basis of the normative model estimated on the NT participants, we first applied spectral clustering with a cosine similarity affinity matrix^52,53^ to the un-thresholded NPMs from the participants with autism.

Next, we applied a multi-class (one-vs-all) linear support vector machine (SVM) to quantify cluster separability within the autism cohort on the basis of the NPMs and perform model order selection (see Supplementary Methods for full details). The number of clusters was determined using the pairwise Area Under the Receiver Operating Characteristic Curve (AUROC) for model orders *K* = 2 to 10. Moreover, we tested the stability of the model using a leave-one-out procedure we have proposed previously^54^.

Next, to evaluate the clinical separability of the clusters, we trained an identical linear SVM to discriminate the clusters using 10 demographic and clinical measures: sex, IQ (verbal, performance and full-scale IQ (VIQ/PIQ/FIQ)), Autism Diagnostic Interview-Revised (ADI-R)^55^ and Autism Diagnostic Observation Schedule (ADOS)-2 calibrated severity scores^56^. Due to the differential availability of measures, we only included participants with data for all clinical measures. This resulted in a decreased sample size of 243 individuals with autism. However, we repeated the analysis with imputed measures, which led to identical conclusions (see Supplementary Methods and Supplementary Table S2). To highlight the important regions for discriminating each cluster we calculated structure coefficients for each class separately^57,58^.

We then assessed associations of each cluster with behavioral measures on the basis of the deviations from the normative model. We first summarized each NPM into a single global measure of deviation—an “atypicality index” for each participant by taking a trimmed mean of 1% of the top absolute deviations across all vertices (Supplementary Methods)^40,59^ and calculated Spearman’s correlation between the atypicality index with ADOS-2 and ADI-R scores, Social Responsiveness Scale-2 (SRS-2)^60^, Repetitive Behavioral Scale-Revised (RBS-R)^61^ and Short Sensory Profile (SSP)^62^, along with the DSM-5 parent-rated scale for attention deficit hyperactivity disorder (ADHD) symptoms (inattention and hyper-impulsivity), both across the whole cohort and within each cluster. To assess the spatial distribution of relevant effects, we also computed atypicality indices for each region after parcellating the cortex using the Desikan–Killiany atlas^63^ and computed correlations with the same measures. See the Supplementary Information for details.

Last, to evaluate the correspondence of the clusters with underlying genetic profiles, we computed the association of the overall atypicality index with polygenic scores (PS) for seven traits (autism, ADHD, epilepsy, FIQ, neuroticism, schizophrenia, and cross disorder risk for psychiatric disorders) using Spearman’s correlation. See the Supplementary Methods for details.

## Results

The average AUROC per model order is shown in Supplementary Fig. S2A indicating that *K* = 5 yielded the highest cluster separability and also was highly stable (Supplementary Fig. S2B).

Table 1 provides the distribution of the clusters in terms of age, sex, and clinical measures. The clusters are evenly distributed across sites, with no obvious evidence for site-related bias (Supplementary Figs. S3 and S4A, B). Cluster 1 contained slightly more females and were slightly younger than the other classes (Table 1). Cluster 2 contained subjects with higher impairment relative to the other clusters, having lower IQ and—relative to some of the other clusters—more severe core autism symptoms across diagnostic instruments (early childhood social symptoms based on ADI-R and current RRB symptoms based on ADOS-2) and ADHD symptoms. None of the other clusters differed in terms of any considered measures.

**Table 1:** Age, sex, and clinical and behavioral scores distribution across clusters.

Measure	Total	Cluster 1	Cluster 2	Cluster 3	Cluster 4	Cluster 5	*P*-value	Post-hoc test
Number of participants [F%]	316 [28%]	60 [43%]	55 [24%]	65 [26%]	55 [25%]	81 [22%]	0.06 (ns)	
Age: mean ± std	17.2 ± 5.7	14.1 ± 5.5	17.3 ± 5.7	17.1 ± 5.7	18.9 ± 5.7	18.2 ± 5.1	<0.001*	1 < 2,3,4,5
IQ								
Verbal IQ	99.6 ± 18.5	106.6 ± 14.7	83.7 ± 16.7	107.4 ± 13.6	96.1 ± 15.9	101.6 ± 19.9	<0.001*	1 > 2,4; 2 < 3,4,5; 3 > 4
Performance IQ	101.7 ± 20.1	104.8 ± 17.7	84.9 ± 19.2	112.3 ± 15.5	100.5 ± 20.4	103.4 ± 18.0	<0.001*	2 < 1,3,4,5; 3 > 4,5
Full-Scale IQ	100.9 ± 18.5	105.9 ± 15.7	83.4 ± 17.1	110.1 ± 13.4	98.2 ± 15.7	103.5 ± 18.3	<0.001*	2 < 1,3,4,5; 3 > 4
ADI-R								
Social	16.2 ± 6.7	15.3 ± 6.4	18.9 ± 6.6	15.4 ± 6.9	15.6 ± 6.4	16.3 ± 6.6	0.03*	2 > 3,1
Communication	13.2 ± 5.7	13.0 ± 4.9	15.3 ± 5.5	12.6 ± 5.8	12.9 ± 6.1	12.6 ± 5.6	0.06 (ns)	
Repetitive behavior	4.3 ± 2.7	4.0 ± 2.8	5.1 ± 2.6	4.5 ± 2.6	3.8 ± 2.1	4.2 ± 2.8	0.09 (ns)	
ADOS-2								
Total	5.2 ± 2.8	4.9 ± 2.8	5.9 ± 3.0	4.7 ± 2.8	4.7 ± 2.3	5.6 ± 2.7	0.08 (ns)	
Social affect	5.8 ± 2.6	5.5 ± 2.7	6.2 ± 2.7	5.6 ± 2.6	5.7 ± 2.4	6.2 ± 2.6	0.52 (ns)	
Repetitive behavior	4.7 ± 2.7	4.7 ± 2.6	5.7 ± 3.1	4.1 ± 2.5	4.0 ± 2.4	5.0 ± 2.6	0.01*	2 > 3,4
SRS-2l								
Score	70.9 ± 11.9	71.8 ± 12.4	72.5 ± 11.1	69.9 ± 11.8	70.1 ± 11.8	70.6 ± 12.0	0.81 (ns)	
SRS-2 self-report								
Score	63.1 ± 10.0	65.8 ± 8.2	68.0 ± 12.0	61.4 ± 9.9	60.2 ± 8.9	62.4 ± 9.3	0.02*	2 > 4
RBS-R								
Score	15.4 ± 13.0	15.5 ± 14.9	20.4 ± 15.7	14.5 ± 10.4	13.1 ± 10.7	14.8 ± 12.1	0.12 (ns)	–
SSP								
Score	140.0 ± 26.2	142.2 ± 25.7	134.2 ± 28.9	144.4 ± 24.7	136.9 ± 25.6	140.1 ± 25.7	0.59 (ns)	–
ADHD								
Hyperactivity/-impulsivity	2.5 ± 2.7	3.1 ± 2.8	3.6 ± 3.0	2.1 ± 2.7	2.3 ± 2.4	2.0 ± 2.4	0.01*	2 > 5
Inattention	4.2 ± 3.1	4.3 ± 3.2	5.2 ± 3.1	3.6 ± 2.9	4.4 ± 3.2	4.0 ± 3.0	0.15 (ns)	–

Cluster differences were assessed via one-way ANOVAs, followed by post-hoc tests to quantify main effects (Tukey Honest Significant Differences). Only for sex distribution across clusters, we used Chi-square statistic. See text for a description of the measures.

*ADI-R* Autism Diagnostic Interview—Revised, *ADOS* Autism Diagnostic Observation Schedule, *RBS-R* Repetitive Behavioral Scale-Revised, *SRS* Social Responsiveness Scale, *SSP* Short Sensory Profile.

^*^P < 0.05.

**Fig. 1:**
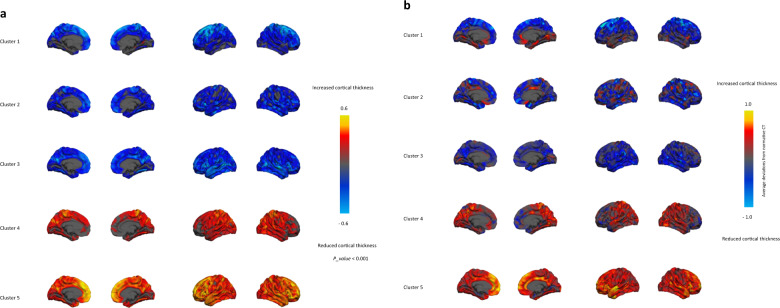
Anatomical separability of the clusters. **a** Structure coefficients. The highlighted regions indicate the importance of each region for multi-class anatomical classification. The positive values are associated with an increased cortical thickness (yellow) and the negative values are in association with the reduced cortical thickness (blue), relative to the other classes. For the purposes of illustration, the structure coefficients were thresholded at *P*<0.001 however this should not be a formal statistical test since these were estimated under cross-validation^68^. **b** The average deviations from normative CT across clusters. Respectively, blue and yellow vertices indicate reduced and increased CT relative to the reference cohort. Maps were rescaled for visualization such that the maximum in each image was = 1.

In terms of anatomical separability, the average pairwise AUROC scores across clusters were above 90% for all clusters (*P* < 0.0001, permutation test). See Supplementary results and Supplementary Fig. S5A, B for more details of anatomical separability performance. Figure 1a shows the structure coefficients indicating the regional differences, which correspond with the mean CT in each cluster shown in Fig. 1b. See Supplementary Fig. S6 for more details. Figure 1a shows striking differences between subtypes: clusters 1, 2, and 3 have reduced CT relative to the expected neurotypical pattern whereas clusters 4 and 5 have increased CT relative to the expected pattern. Clusters also varied in terms of spatial distribution. For instance, clusters 5 and 4 had increased CT in the prefrontal and posterior parietal cortex, respectively, while cluster 3 had reduced CT in temporal gyrus and cluster 1 shows decreasing CT in the anterior premotor cortex.

**Fig. 2:**
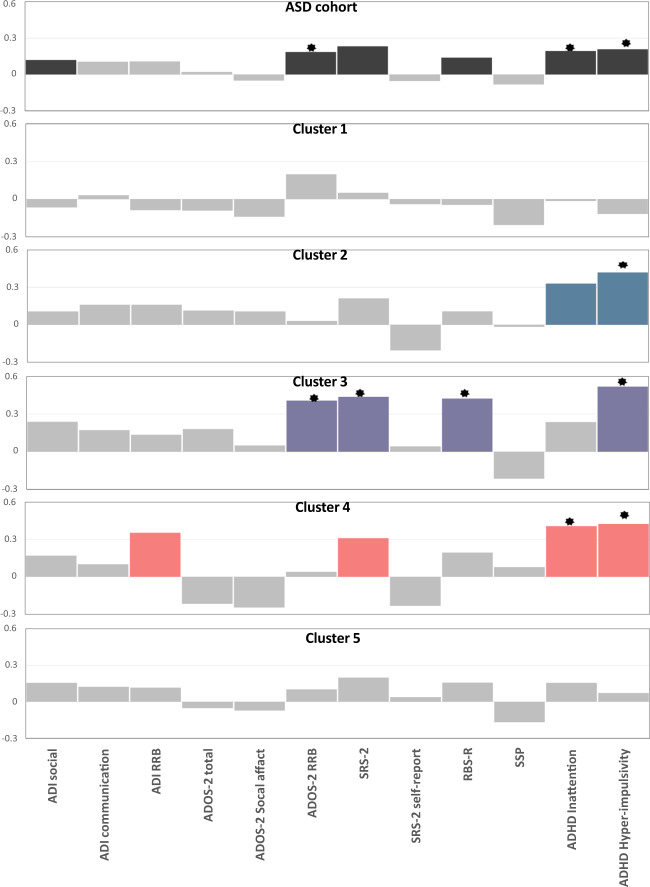
Correlation of atypicality index with symptoms. Colored bars show a correlation with *P* < 0.05. * indicates significant correlations after FDR correction across clusters and blocks. Note that the *y* axis is fixed across all panels. Autism Diagnostic Interview-Revised (ADI-R), Autism Diagnostic Observation Schedule (ADOS)-2 calibrated severity scores, Social Responsiveness Scale-2 (SRS-2), Repetitive Behavioral Scale-Revised (RBS-R), Short Sensory Profile(SSP), DSM-5 ADHD rating scale for attention deficit hyperactivity disorder (ADHD) symptoms (inattention and hyper-impulsivity). The ADHD scores are parent-report scores.

Figure 2 shows the correlation of behavioral measures with the atypicality index across the cohort and per cluster, corrected within symptom domains and across clusters. While the top panel indicates only moderate correlations of several behavioral measures with the atypicality index across the cohort, the magnitude of correlations increased for nearly all measures when considering them by cluster and additional associations reached significance for some measures (e.g., ADI-RRB). This provides evidence that clinical associations are diluted across the cohort. The only exception to this pattern was the association with ADI-social, which reached nominal significance across the entire sample but not in any subtypes.

Moreover, the classifier trained on the clinical and behavioral scores also discriminated the clusters above chance level under cross-validation (accuracy = 0.32 chance = 0.20, AUROC scores = 0.58, *P* < 0.05, permutation test). See Supplementary Results and Supplementary Fig. S7A, B.

**Fig. 3:**
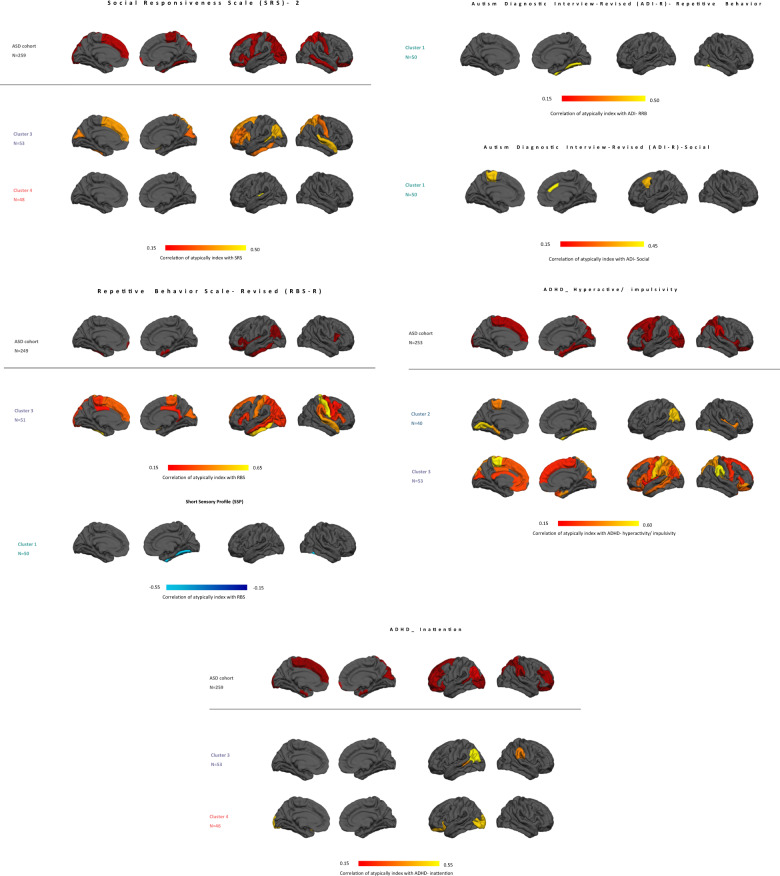
Regional atypicality index associations with symptoms. Only regions surviving FDR correction (*q* < 0.05) are shown. Note that ADHD scores are parent-reported scores.

The regional associations with symptoms are shown in Fig. 3. These show a similar pattern in that the different clusters are associated with similar symptoms and in nearly all cases associations become stronger for each cluster relative to across the cohort. This again indicates that clinical effects are diluted across the whole cohort relative to within clusters: for parent-reported measures of ADHD, social-communicative symptoms (SRS-2) and restricted and repetitive behaviors (RBS-R), the correlation becomes stronger within the cluster and the association with early childhood repetitive behaviors and social symptoms (ADI-R RRB and social) exclusively appears within the clusters. Finally, cluster 1 also shows focal associations with sensory atypicalities and early childhood repetitive behaviors, which were not evident at the whole-brain level.

Finally, the association of the atypicality index with polygenic scores ( Supplementary Fig. S8) shows a similar pattern, namely that associations become stronger within the clusters, but in this case, the only cluster 3 was associated with polygenic risk for autism and schizophrenia.

## Discussion

In this study, we aimed to understand the biological heterogeneity of autism by identifying potential neuroanatomical subtypes within a large and well-phenotyped cohort. To do so, we employed spectral clustering on the individual-level deviations from a normative model of CT development. Normative modeling learns supervised mappings that describe cortical development, also accounting for sex and nuisance variation. This allows the clustering algorithm to focus upon clinically relevant variation better than by clustering the data directly and therefore more precisely dissect biological heterogeneity^40,64^. Through this, we identify five putative neuroanatomical subtypes within the cohort that: (i) show striking differences in that some subtypes have reduced cortical thickness relative to the neurotypical pattern and others have increased thickness; (ii) could be accurately classified from one another out of sample; (iii) predict differential symptom profiles; (iv) load differentially onto underlying genetic risk and most importantly, (v) are more strongly associated with symptoms than associations across the whole cohort. Taken together, our results show that aggregating effects across individuals dilutes the strength of clinically relevant associations and by stratifying individuals according to their underlying biological signature it is possible to reduce the heterogeneity in clinical presentation.

Among the five subtypes, three showed widespread decreased CT, and two showing increased cortical thickness reflecting ~57% and 43% of the cohort, respectively. These distinct and opposing expressions of CT atypicalities across different individuals with autism provide an explanation of why classical case–control analyses provide inconsistent findings and why they often reveal no or only modest group differences^42,46^.

Sub-dividing individuals with autism into more homogenous subtypes has important clinical implications. Specifically, even though clusters of different CT profiles are solely based on imaging phenotypes, they can still be discriminated based on clinical profiles. Only cluster (1) was weakly associated with age and there were moderate differences in IQ between clusters but no strong association with symptoms. Together this suggests that subtypes reflect distinct cortical fingerprints rather than different neurodevelopment stages or simply different symptom groups. It is increasingly well recognized that there is no reason to believe that distinct biological subtypes will map cleanly onto different symptom groups because distinct pathophysiological pathways likely converge on the same symptoms^5,21^. Therefore, we consider that comparing the strength of associations between biological readouts and clinically relevant variables within clusters rather than across the cohort provides a better evaluation of whether the clusters explain clinically relevant mechanisms than testing for mean differences in clinical variables between clusters. More specifically, because there is no one-to-one mapping between biology and symptoms, the increased homogeneity within a cluster does not necessarily translate into increased homogeneity of the clinical profile. Also, it should be remembered that the patterns of deviation are complex and for the purposes of computing clinical associations, we summarize these patterns in an atypicality index, which is a single number describing maximum overall deviation. Other indices are also possible, which may reflect different features of the underlying patterns of deviation. In addition, and in contrast with recent suggestions that the heterogeneity underlying autism is not a biological fact of nature^18,65^, our results suggest that with the right analytical approach, biological heterogeneity is not only quantifiable but also leads to a stratification of individuals having more consistent biological profiles than can be detected by case–control comparisons. Our results indicate those inconclusive findings in the autism literature may be the result of the approaches that assume the autism cohort is well-defined and homogeneous.

Among the clusters we have reported, three (clusters 2, 3, and 4) demonstrated specific patterns of alterations relevant to symptoms at the whole-brain level and cluster 1 additionally showed regionally specific associations. Among core autism symptoms, the associations were most prominent with repetitive behaviors.

Cluster 3 (~21% of individuals with autism), was the only cluster that showed associations with core autism symptoms (i.e., RBS, SRS, and ADOS-2-RRB) plus hyperactivity-impulsivity and was also the only cluster to show associations with polygenic risk. Neuroanatomically, cluster 3 involves dorsal frontal eye fields plus anterior cingulate, premotor, auditory, and temporal cortices (Fig. 1). Taken together, this suggests this cluster represents patients with a broad clinical phenotype who were impaired across multiple domains combined with prominent alterations in patterns of CT.

Cluster 2 (17% of individuals with autism), was associated with comorbid ADHD symptoms and neuroanatomically widespread cortical thinning prominently in the dorsolateral and anterior prefrontal cortex, orbitofrontal area, dorsal anterior cingulate cortices, primary somatosensory cortex, superior parietal lobule, primary motor cortex, temporal gyrus, and Broca’s area (Fig. 1). Alterations in similar brain regions have previously been reported in individuals with ADHD^66,67^. This points to overlap of the pathophysiological pathways underlying autism and ADHD.

Cluster 4 (17% of participants with autism), was also most strongly associated with inattention and hyper-impulsivity but also showed nominally significant associations with SRS and ADI-R-RRB, although these did not survive multiple comparison correction. The anatomical profile of cluster 4 exhibits cortical thickening mainly in the primary somatosensory and motor cortex, superior parietal lobule, associative visual cortex and, frontal eye fields. Associations with ADI-social scores (even though weak) only emerged across the entire sample (i.e., that was not also significant for some of the subtypes), potentially implying that social deficits are a unifying feature across all individuals with autism.

Many studies have focused on finding subtypes based on behavioral and clinical measures^22,29–31^. As an example, Fountain et al.^32^ reported six subtypes on the basis of social, communication, and repetitive behavior functioning scores. While this is intuitively appealing, there is no guarantee that such subtypes map onto neurobiologically distinct phenotypes. Indeed, our results show that highly different patterns of atypicality are associated with symptoms across the cohort, which illustrates why clustering on the basis of symptoms may not yield biologically relevant stratifications. In contrast, Hong et al.^24^ developed an approach to multidimensional neuroimaging data. Other approaches^23–26,32,33^, similarly aimed to biologically stratify autism using raw data features (i.e., without considering individualized alterations from a normative pattern). We consider that the approach we have employed (i.e., finding consistent patterns of individualized variation from a normative pattern) leads to more interpretable clusters than clustering the data directly since it accounts for multiple sources of variation. Another feature of our approach is that it capitalizes upon both dimensional and categorical aspects of the neurobiology of autism. Specifically, the normative model captures dimensional variation to provide an optimal representation for finding categorical differences (i.e., clusters). This is complimentary to a recent study^27^, which also adopted a dimensional approach by estimating potentially overlapping autism-related latent factors learned from functional connectivity data. While a direct comparison of these different analytical approaches is beyond the scope of the present work, in our data, it is salient that in our data the clusters had very low overlap with one another. More generally, finding latent factors is well suited to finding latent profiles that overlap across subjects, whereas clustering is suited for finding subjects that share a common profile. Despite strong anatomical separability and behavioral associations within the clusters we report, we are cautious about claiming that there are definitively five subtypes of autism because we consider that requires further validation than was undertaken here. For example, it will be important to validate these findings across additional cohorts and in terms of the ability to predict the clinical outcomes or additional biological or phenotypic measures. Rather, we are using clustering as a tool to fractionate the clinical phenotype of autism on the basis of the underlying biology and to understand variation across clinically realistic cohorts. An additional important consideration that we have identified previously is to test against the ‘null’ distribution of no clusters in the data^54^. This was not feasible in this study because of the high dimensionality of the clustering problem (i.e., making it difficult to sample realistic ‘null’ biological patterns). However, the fact that we have stronger associations with behavior in many distinct clinical measures, as well as polygenic scores relative to across the whole cohort strongly suggests that the clustering is more meaningful than a simple continuum. Assessment of other LEAP data modalities (e.g., EEG, eye-tracking, diffusion-weighted imaging) will be the subject of future studies.

There is a number of limitations associated with the current study. Firstly, there was a large number of missing clinical and behavioral data, which complicated analyses and led to a reduction of power in follow-up analyses. Most importantly, for the genetic analysis, the interpretation of the results should be made in the context of relatively low statistical power for each class and should be considered illustrative until our findings can be validated in larger cohorts. It is also possible that data were not missing at random. Secondly, regarding the anatomical data, we did not perform any manual edits on the cortical surface reconstructions. While this eliminates one potential source of operator bias, the quality of the surface reconstructions could be improved in some cases by performing manual edits.

In conclusion, we identified several putative autism subtypes across a highly heterogeneous cohort. These were highly anatomically distinct and showed stronger clinical, behavioral, and genetic associations than across the whole cohort. This is a promising step towards stratification tools and a better understanding of the heterogeneous neurobiology of autism.

## Supplementary information

Supplementary Information
