# Supplementary material for: Fractionating autism based on neuroanatomical normative modeling
Source: Transl Psychiatry. 2020 Nov 6;10:384. doi: 10.1038/s41398-020-01057-0 (PMC7648836; doi:10.1038/s41398-020-01057-0)
Supplement: Supplementary file 1 — Supplementary Information [file 41398_2020_1057_MOESM1_ESM.docx]

**Fractionating autism based on neuroanatomical normative modeling**

***Supplementary Information***

**Supplementary Methods**

**Sample**

Here we included 206 neurotypical (NT) (79 female, aged 17.5 ± 6.1 years) and 316 participants with autism (88 female, aged 17.2± 5.7) across 6 sites from the EU-AIMS Longitudinal European Autism Project (LEAP) sample ^1^. In order to obtain a normative reference, the participants with Intellectual disability were excluded from the NT cohort. Table S.1 shows the clinical characteristics of participants with autism across different sites. Following the standard neuroimaging contraindications, individuals with claustrophobia or metal implants were excluded in addition to the Individuals who had a history of bipolar disorder or psychosis. Here, the emphasis of the study was realistically characterizing the heterogeneity within autism spectrum disorder (ASD) at the level of the individual. Among the patients with autism, up to 70% of ASD individuals have one or more psychiatric conditions ^2^ and 30-50% of individuals with ASD are on stable medications ^3^. Hence, in contrast to most case-control studies, we did not expand the exclusion criteria towards other comorbidities (e.g. attention deficit/hyperactivity disorder) to create a realistic autism cohort.

**Participants**

Participants with autism were recruited from existing databases and clinic contacts across one of six study sites: the Institute of Psychiatry, Psychology and Neuroscience, King’s College London, London, United Kingdom; Autism Research Centre at the University of Cambridge, Cambridge, United Kingdom; Radboud University Nijmegen Medical Centre, Nijmegen, the Netherlands; University Medical Centre Utrecht, Utrecht, the Netherlands; Central Institute of Mental Health, Mannheim, Germany; and University Campus Bio-Medico, Rome, Italy. Table S.1 shows the clinical characteristics of participants with autism across different sites.

Table S.1: Autism participants’ characterization: The mean and standard deviation (SD) of age, intelligence quotient (IQ), Autism Diagnostic Observation Schedule (ADOS), Autism Diagnostic Interview-Revised (ADI-R) and number of participants in each sites. * indicates P<0.05 in ANOVA.

| **variable** | | **Cambridge** | **KCL** | **Mannheim** | **Nijmegen** | **Rome** | **Utrecht** |
| --- | --- | --- | --- | --- | --- | --- | --- |
| **Age, mean, [SD]** | | 17.6 [5.8] | 17.0 [5.8] | 16.0 [2.8] | 16.2 [5.6] | 24.9 [2.9] | 16.8 [5.5] |
| **IQ** | |  |  |  |  |  |  |
|  | Full-scale IQ | 106 [19] | 100 [21] | 102 [13] | 97 [18] | 101 [14] | 105 [13] |
|  | Performance IQ * | 109 [21] | 99 [20] | 105 [13] | 97 [22] | 104 [18] | 107 [17] |
|  | Verbal IQ | 103 [17] | 99 [20] | 101 [15] | 97 [19] | 98 [16] | 105 [14] |
| **ADI-R[SD]** | |  |  |  |  |  |  |
|  | Social* | 17.2 [6.6] | 18.2 [6.2] | 15.1 [7.5] | 14.4 [6.4] | 11.4 [5.7] | 16.3 [5.8] |
|  | Communication* | 14.5 [2.7] | 15.3 [5.3] | 10.6 [5.0] | 12.7 [5.4] | 9.3 [5.4] | 11.3 [5.4] |
|  | Repetitive Behavior* | 5.0 [2.7] | 5.0 [2.4] | 4.8 [3.6] | 2.9 [2.1] | 5.3 [2.3] | 3.5 [2.7] |
| **ADOS [SD]** | |  |  |  |  |  |  |
|  | Total | 5.2 [2.4] | 5.1 [2.9] | - | 5.3 [2.7] | - | 4.8 [2.7] |
|  | Social* | 6.3 [1.8] | 5.3 [2.8] | - | 6.1 [2.5] | - | 5.5 [2.7] |
|  | Repetitive Behavior* | 4.5[2.5] | 5.7 [2.6] | - | 3.7[2.7] | - | 4.3[2.4] |
| **TOTAL** | | 40 | 119 | 26 | 80 | 17 | 34 |

**Magnetic Resonance Imaging**

A high resolution T1-weighted image was acquired from each participant with a standard Alzheimer’s Disease Neuroimaging Initiative (ADNI) sequence ^4^, matched across scanning sites. Cortical thickness was estimated from the high-resolution T1-weighted image for each subject using FreeSurfer version 5.3 (<http://surfer.nmr.mgh.harvard.edu/>). Prior to analysis, all surface reconstructions were visually assessed for reconstruction errors by at least 3 independent raters. We excluded a small number of scans with severe artifacts (e.g. caused by head motion). The rest of the scans were included ‘as is’, i.e. we did not allow manual edits to reduce the possibility of bias (e.g. due to individual differences in operator skill). Cortical thickness maps were then smoothed with a 10-mm surface-based Gaussian kernel.

**Normative modelling**

Gaussian process regression (GPR) ^5^ was used to estimate a normative model of CT across the cortical surface ^6,7^. GPR was trained on the NT cohort (N=206) using the following covariates (i.e. independent variables): age, sex, full-scale IQ (FIQ), the Freesurfer Euler number ^8^ indicating the topological complexity of the reconstructed cortical surface and which has been validated as a surrogate marker for image quality ^9^, plus dummy coded variables representing data acquisition sites. These covariates were used to predict CT as a response (dependent) variable ^6^. We used 10-fold cross-validation during the training process to provide an unbiased estimation of the model.  Essentially, normative modeling presents a probabilistic interpretation of the deviations across all subjects. To quantify the pattern of deviation from the normative modeling for vertex-wise CT, we defined a normative probability map (NPM). The NPM provides a statistical estimate of an individual deviation from the neurotypical pattern at each vertex. To construct the NPM, we used the predicted CT of the normative model at each vertex and for each individual participant, and next converted each to a subject-specific *Z* score as described previously ^6^. We then created/calculated NPMs for participants with autism, which quantify the deviation of regional CT from the expected neurotypical pattern (N=316).

**Imputed data**

Table S.2 shows the subset of behavioral measures with at least one missing data value. Here, missing values were imputed using multivariate regression models as implemented in^10^. Briefly, for a chosen regression model, a round-robin regression approach is used where every variable is considered as an output in turn. Variables are imputed according to an increasing number of missing values, and variables imputation is repeated cyclically till convergence. To optimize the imputation, we evaluate different regression models including Extra Trees , K-Nearest Neighbors, and Bayesian Ridge regression; for comparison, we also considered two common simpler strategies, mean and median variable imputation. To evaluate each model, we performed a leave-one-observation-out cross-validation approach where at each fold, we added one extra missing value to the original data by removing an actual observation before fitting the regression model. Then, at each fold, we evaluate the model performance by computing the squared error at the artificially added extra missing value. Statistics over folds showed that all regression models outperform mean and median imputation and that Extra Ridge Regression and Decision Trees were the best models for most variables (Table S.2)

Table S.2: List of imputed measures

| Measure | Regression model |
| --- | --- |
| ADI-R: |  |
| Social | Bayesian Ridge |
| Communication | Bayesian Ridge |
| Repetitive behavior | K-Nearest Neighbors |
| ADOS-2: |  |
| Total | Extra Trees |
| Social affect | Extra Trees |
| Repetitive behavior | Extra Trees |
| SRS-2l: | Bayesian Ridge |
| SRS-2 self-report: | Bayesian Ridge |
| RBS-R: | Extra Trees |
| SSP | Bayesian Ridge |
| ADHD: |  |
| Hyperactivity/-impulsivity | Extra Trees |
| Inattention | Bayesian Ridge |

**Clustering**

A methodological overview of our approach is shown in Figure S.1. We first applied spectral clustering ^11,12^ to the un-thresholded normative probability maps (NPMs), describing the deviations from the normative model in order to partition the autism cohort into sub-clusters on the basis of the normative model estimated on the NT participants. We employed 10,000 random initializations and selected the best output of consecutive runs in terms of minimizing the sum of squared errors for each cluster (i.e. ‘inertia’) as the final model, indicating the most compact and dense clusters across other models. To define the affinity matrix, we measured the similarity of autism deviating maps using cosine similarity, which is a well-suited measure for high-dimensional spaces ^13^.

Next, we developed an approach to evaluate the separability of the clusters and to assist with model order selection (i.e. the number of clusters) where we applied a multi-class (‘one-vs-all’) linear support vector machine (SVM) on the NPMs. The SVM regularization parameter was fixed to C=0.25 for all analyses and prior to applying SVM, the dimensionality of the NPMs was reduced to 10 components using principal component analysis (PCA). The number of clusters was determined using the pairwise Area Under the Receiver Operating Characteristic Curve (AUC ROC) scores for model orders K=2 to 10. Moreover, we tested the stability of the model using leave-one-out procedure ^14^.

To evaluate the clinical utility of the clusters, we investigated associations of clinical scores with our anatomically identified clusters using an identical linear SVM architecture. We selected 10 demographic and clinical measures –which had the least number of missing data across all the cohort- comprising sex, IQ measures (verbal IQ (VIQ), performance IQ (PIQ) and full-scale IQ (FIQ)), and autism symptom scores, namely the Autism Diagnostic Interview-Revised (ADI-R) ^15^ and Autism Diagnostic Observation Schedule (ADOS)-2 calibrated severity scores ^16^ sub-scores. Due to differential availability of measures across the autism cohort, we only included the participants with available data for all the clinical measures. This resulted in a decreased sample size of 243 individuals with autism. However, we repeated the analysis with imputed measures to compensate for the large number of missing data, which led to identical conclusions.

The varying sample size across sub-clusters leads to classification problems with imbalanced classes. To tackle this problem, we artificially balanced classes using up-sampling. In every iteration, we split each class into test and training sets and randomly assigned N_test=5 samples per class to the test set. We drew (N_training=45) training samples randomly from the remaining samples in the class if the remaining sample size was greater than N_training, otherwise, the remaining samples were added to the training set and then we up-sampled the data from the remaining samples until it reached N_training.

To highlight the important regions for the classifiers in each cluster we calculated the structure coefficients using the method described in ^17^ for each class separately. Briefly, we mapped the covariance of the variables set (clinical and behavioral measures or CT deviating map) to input space. To better interpret these regions, we calculated the structure coefficients by computing the Pearson correlation of X and the linear predictor (XW) for each cluster, where X is the vector of covariates and XW is the inner product of X and the weights of each class of the classifier^17,18^.

Further, we assessed the associations of each cluster with behavioural measures on the basis of the deviations from the normative model. The normative model provides a multivariate measure of deviations for each subject. In order to summarize the NPM into a single measure (‘atypicality index’) we computed a 10% trimmed mean of the 1% top absolute deviations for each subject across all vertices ^6,7^. The atypicality index provides a summary of deviation at the individual level. To assess the behavioral relevance of the deviations across these clusters, we calculated Spearman’s correlation coefficients between overall and regional extreme deviations from the normative model (an ‘atypicality index’) and behavioral scores (*p* < 0.05, FDR) both across the whole cohort and within each cluster. To assess the spatial distribution of relevant effects and similar to the overall atypicality index, we also computed regional extreme deviations as the trimmed mean of the 1% of top absolute deviations for each region after parcellating the cortex using the Desikan-Killiany atlas ^19^. We then estimated correlations between the regional deviations and a number of autism-related measures, namely the ADOS-2 and ADI-R, Social Responsiveness Scale-2 (SRS-2) ^20^, Repetitive Behavioral Scale-Revised (RBS-R) ^21^ and Short Sensory Profile (SSP) ^22^, along with the DSM-5 ADHD rating scale for attention deficit hyperactivity disorder (ADHD) symptoms (inattention and hyper-impulsivity). Due to availability of scores, we only used parent-reported scores for ADHD measures. See the supplementary method for details. These analyses were performed across the entire cohort and within each cluster.

**Genotyping**

To evaluate the correspondence of the neurobiologically defined clusters with underlying genetic profiles, we computed the association of the overall atypicality index with polygenic scores (PS) for 7 traits (autism, ADHD, epilepsy, intelligence (FIQ), neuroticism, schizophrenia, and cross disorder risk for psychiatric disorders).

The genotyping was performed at the Centre National de Recherche en Génomique Humaine (CNRGH) using the Infinium OmniExpress-24v1 BeadChip (> 700K markers) from Illumina. Sample quality controls such as Sex check (based on the X chromosome homozygosity rate or the median of the Log R ratio of the X and Y chromosomes), Mendel errors (transmission errors within full trios) and Identity By State (IBS, see section below) were performed using PLINK 1.90. Imputation of 17 million SNPs was performed using the 700k genotyped SNPs on the Michigan Imputation Server ^23^.We used the HRC r1.1 2016 reference panel for a European population because the majority of individuals in the LEAP cohort were from European ancestry. It is to be noted that only autosomes are imputed, the sex chromosomes are present in the non-imputed files.

**Ancestry**

Principal components analysis (PCA) of a variance standardized relationship matrix was used to evaluate the ancestry of individuals in LEAP cohort and to provide components for any covariate adjustments. To go further and to cluster individuals based on ancestry, we further reduced dimensionality with uniform manifold approximation and projection (UMAP; ^24^) reducing the first 8 PCA components to 2 components (x-umap-spread and y-umap-spread columns) that allows for better visualization and easier interpretation. Finally, to find subpopulation clusters (hdbscan column), weperformed density based clustering (HDBSCAN; ^25^).

**Inbreeding**

For the estimation of the inbreeding coefficient, SNPs with genotyping call rate < 95%, minor allele frequency < 0.05, strong linkage disequilibrium r > 0.5 or failing Hardy Weinberg equilibrium test (p < 10-6) were filtered out of the SNP genotyping dataset. All homozygosity analyses were performed with Plink 1.09 on autosomes including identification of Runs Of Homozygosity (ROH) and Inbreeding coefficients calculation. For ROH detection, a threshold of 50 consecutive homozygous SNPs with a minimum density of 1 SNP / 5,000 kb and no minimum length was used following ^26^. We allowed no heterozygous markers in the 50 SNPs-window. Inbreeding coefficients were calculated by estimating the proportion of the autosomal genome that is in ROH (FROH column).

**Estimation of polygenic scores**

The Polygenic Scores (PSs) were computed on 343 individuals with autism (including individual with ASD and intellectual disability (ID)) and 241 typically developing controls (TD) using PRSice-2 tool (PRSice: ^27^. For each PS, a GWAS summary statistics was used as a reference (all with an additive model) and for the linkage disequilibrium-based SNP pruning only the SNP with a minor allele frequency>1% and with a R2 < 0.1 in windows of 500kb were selected. PS were adjusted for principal component ancestry using PC1 to PC4. The computed PSs are summarized below, along with a reference for the GWAS used.

1. Attention Deficit and Hyper activity Disorder (PS-ADHD) ^28^
2. Autism Spectrum Disorder (PS-ASD_ipsych) ^29^
3. Epilepsy (PS-Epilepsy) ^30^
4. Intelligence (PS-Intelligence) ^31^
5. Neuroticism (PS-Neuroticism) ^32^
6. Schizophrenia (PS-SCZ2) ^33^
7. Psychiatric cross-disorders (PS-xDx_ipsych) ^34^

**Methodological overview**

A methodological overview of the approach is shown in Figure S.1.

Figure S.1: The methodological overview of the approach


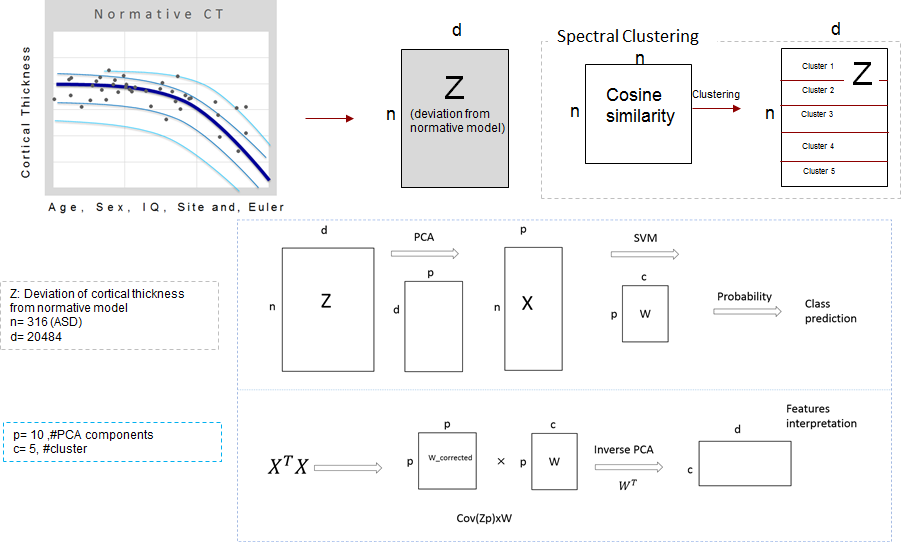


**Supplementary Results**

**Model order**

To determine the number of clusters of spectral clustering approach, we tested the anatomical separability for model orders K=2 to 10. The best K in terms of separability indicates the number of clusters. Figure S.2.A shows the average of pairwise Area Under the Receiver Operating Characteristic Curve (AUC ROC) scores across evaluated model orders. The confidence intervals were calculated using Hanley and McNeil approach ^35^. Figure S.2.B shows the stability of the model with K=5 for 50,000 runs. In each run, the similarity between two clusterings were measured using adjusted rand index by considering all pairs of samples and counting pairs that are assigned in the same or different clusters. Note that these two clustering excluded sample *i* and *j* where $i\neq j$.

Figure S.2.A: Determining the spectral clustering model order using AUC ROC scores for K=2 to 10. The confidence intervals calculated using Hanley and McNeil approach ^35^


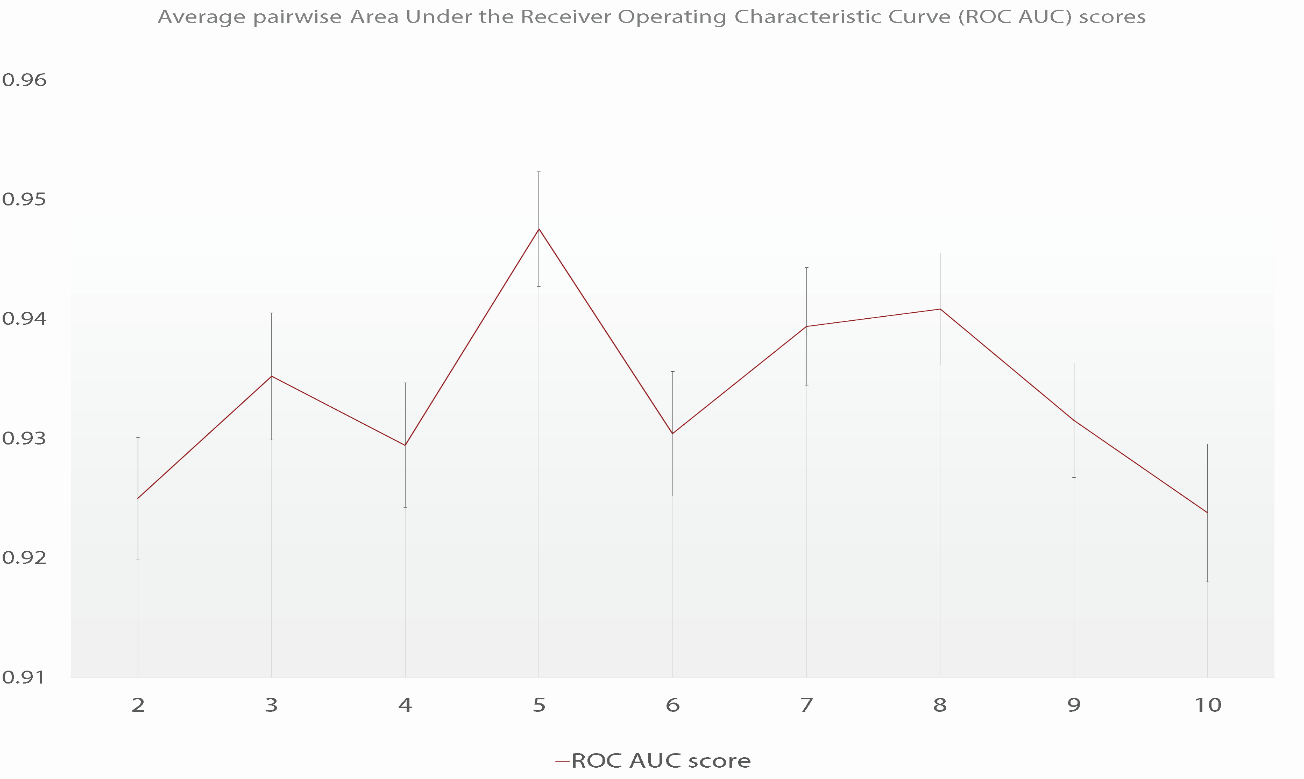


*Figure S.2.B: Adjusted rand score over 50,000 runs.*  **
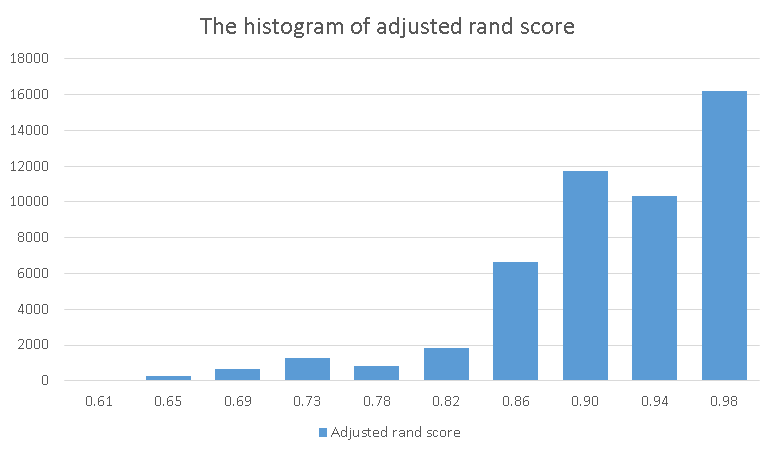
**

**Clusters characterization**

Figure S.3 shows the distribution of sites across clusters.

Figure S.3: Distribution of sites across clusters

**
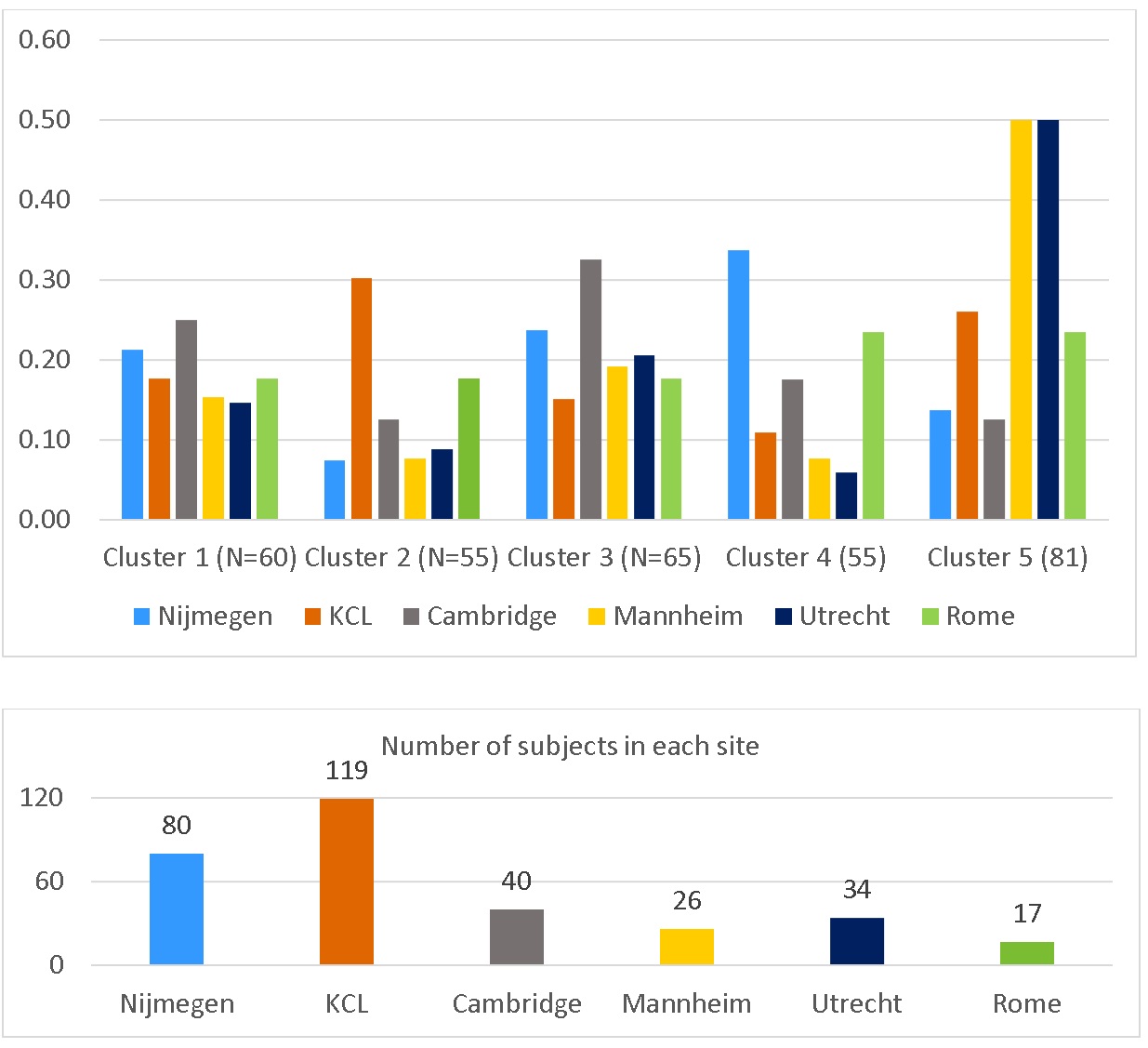
**

Figure S.4.A and Figure S.4.B show the distribution of age and sex across clusters, respectively.

Figure S.4.A: Age distribution across clusters

**
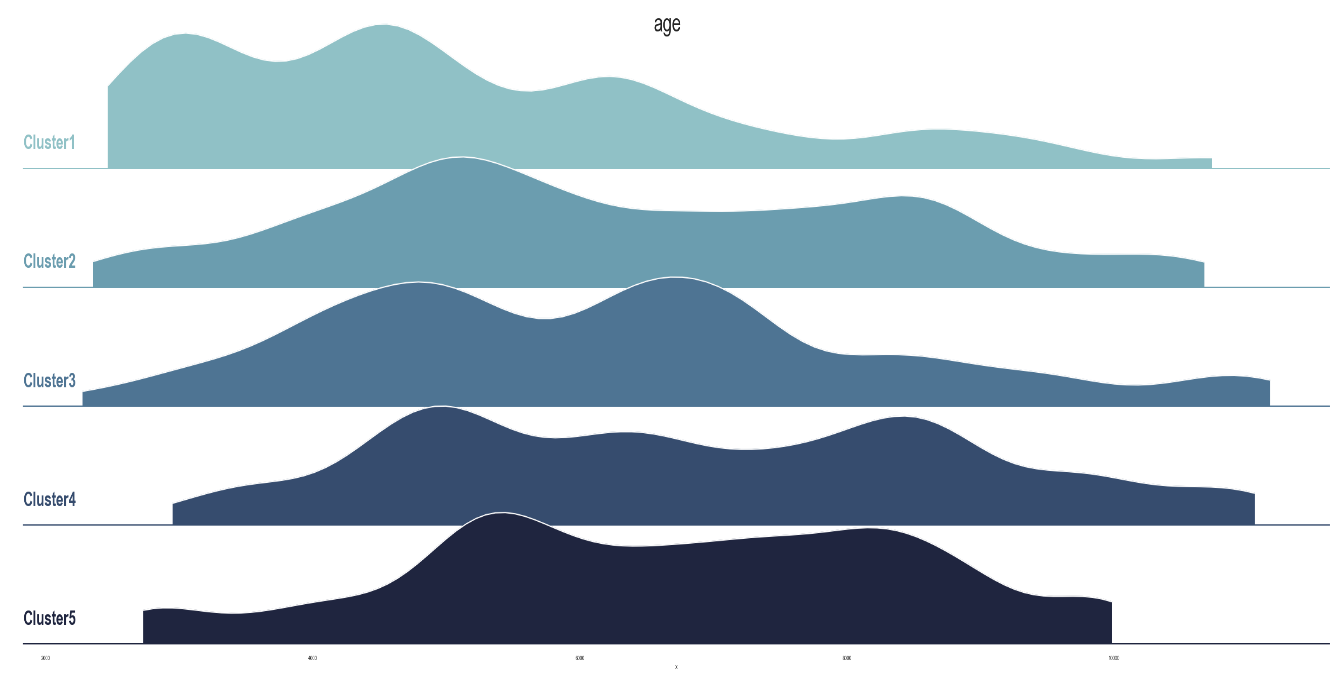
**

Figure S.4.B: Sex distribution across clusters


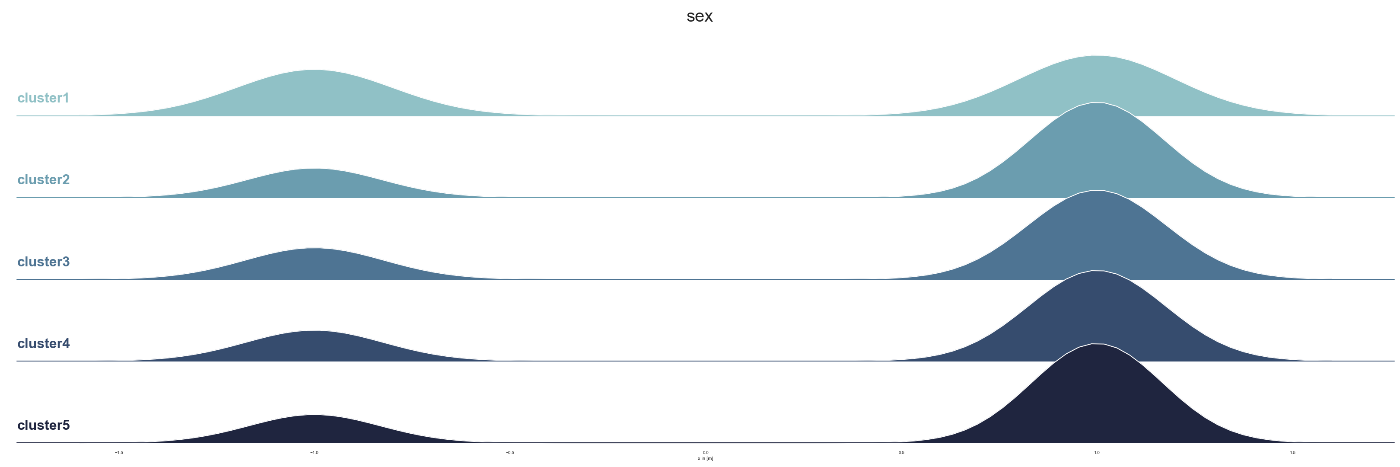


**Anatomical separability**

The anatomical separability of the clusters was tested using a linear support vector machine (SVM) with nested cross-validation (number of iteration= 10,000). Figure S.5.A and Figure S.5.B show the accuracy confusion matrix and the average pairwise AUC ROC scores across clusters, respectively (all the scores significant, P-value <0.0001, permutation test).

*Figure S.5.A: Anatomical separabilty confusion matrix*


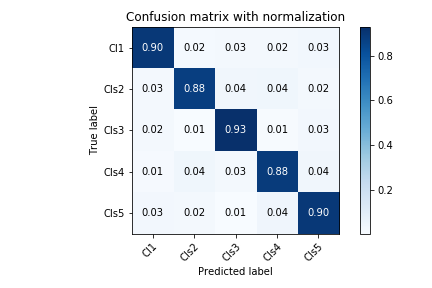


Figure S.5.B: Anatomical separability AUC ROC scores across clusters


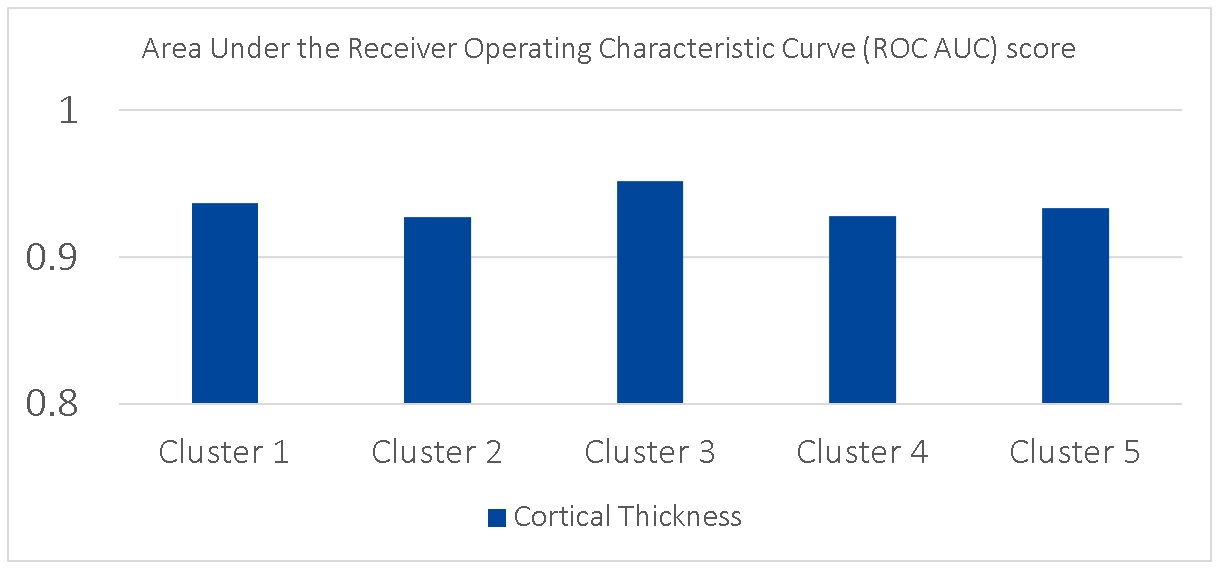


Figure S.6: The average of positive and negative deviations across each cluster. Respectively, Blue and yellow vertices indicate reduced and increased CT related to the reference cohort.

**

**

**

**

**Clinical separability**

Figure S.7.A shows the separability of clinical measures in terms of ROC AUC scores. The structure coefficients for clinical measures shown in Figure S.7.B indicates that IQ measures contribute the most overall. Further, ASD-related scores contribute moderately in cluster 2 and cluster 3. From another point of view, it may well be argued there is a reverse relationship between IQ measures and ASD-related measures except for ADI-RRB in the first three clusters, which alternates in a similar manner to the IQ measures.

Figure S.7.A: Anatomical separability AUC ROC scores across clusters for Clinical and behavioral measures (i.e., sex, IQ, ADOS and, ADI)


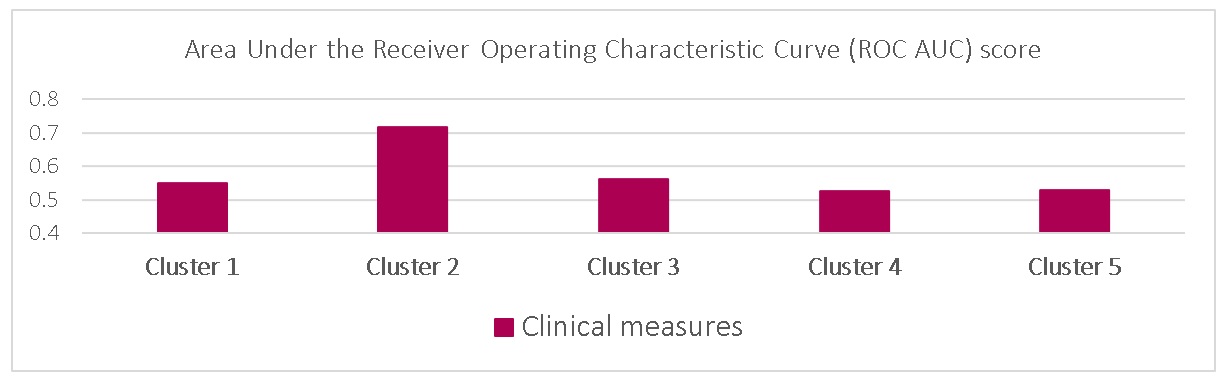


Figure S.7.B: Structure coefficients (clinical measures)

**
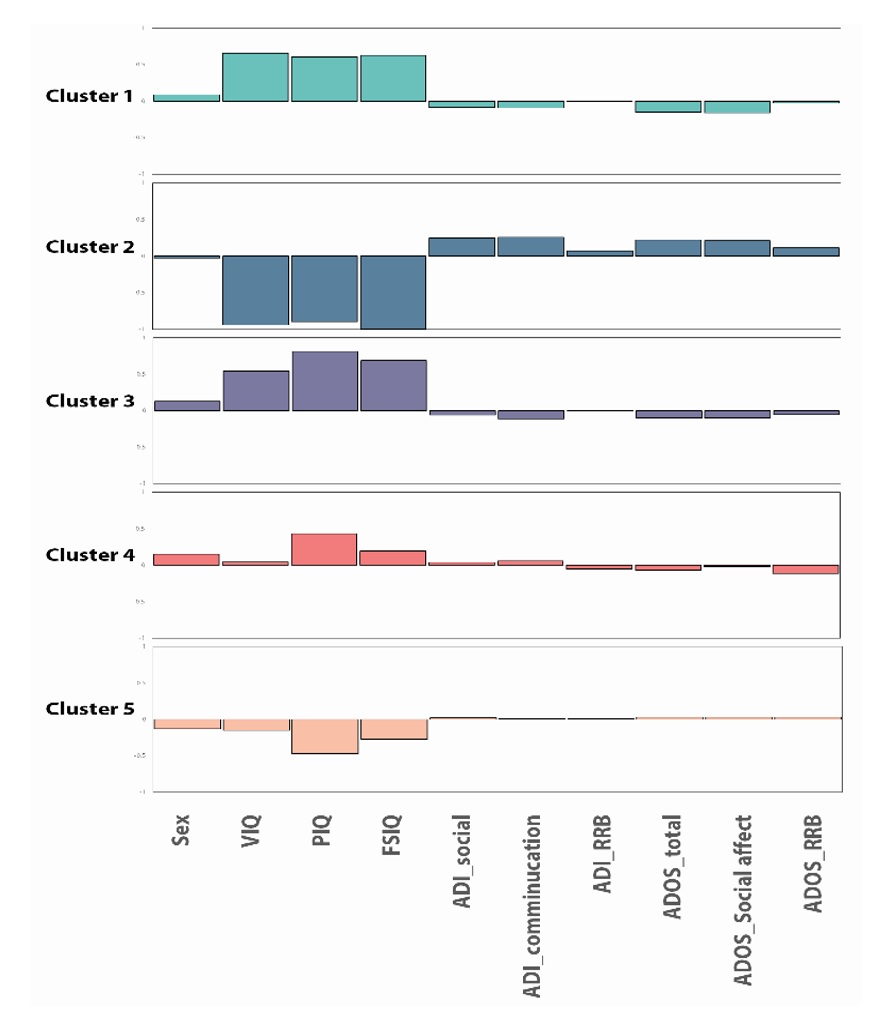
**

**Polygenic score association**

Figure S.8: Correlation of atypicality index with polygenic scores. Colored bars show correlation with P <0.05. * indicates the significant correlations after FDR correction across clusters


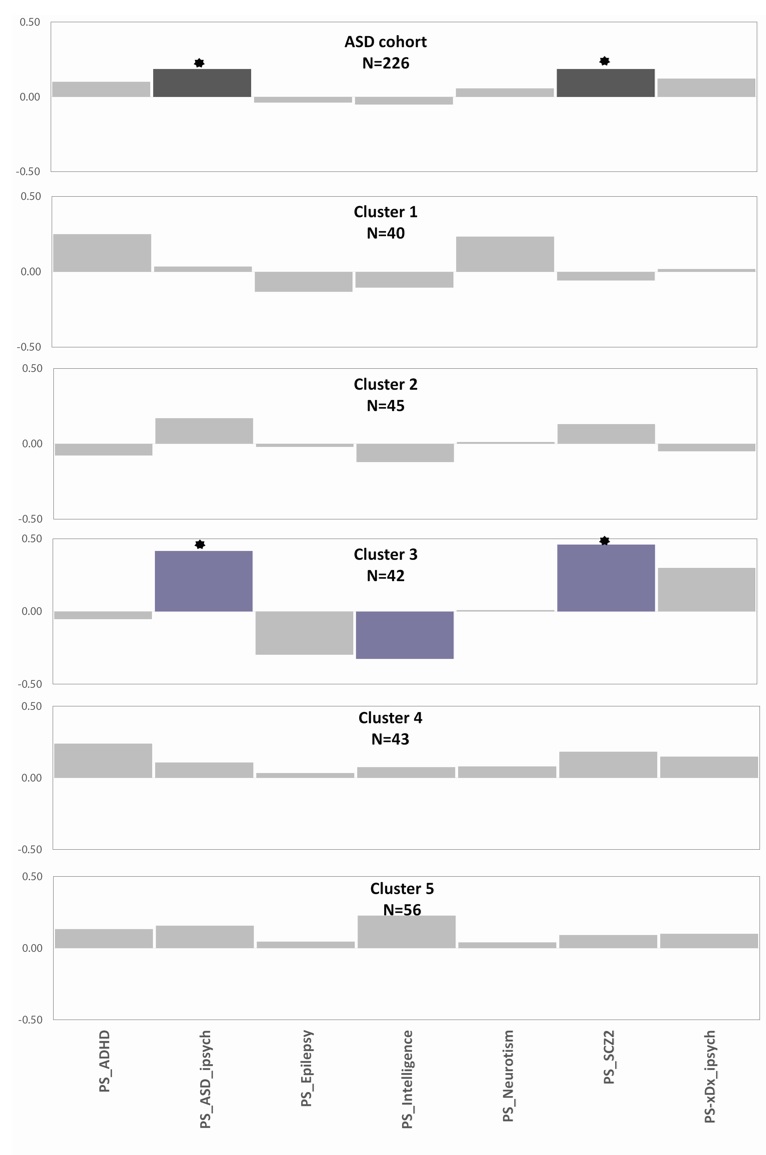


**Supplementary References**

1. Charman, T. *et al.* The EU-AIMS Longitudinal European Autism Project (LEAP): clinical characterisation. *Mol. Autism* **8**, 27 (2017).

2. Simonoff, E. *et al.* Psychiatric Disorders in Children With Autism Spectrum Disorders: Prevalence, Comorbidity, and Associated Factors in a Population-Derived Sample. *J. Am. Acad. Child Adolesc. Psychiatry* **47**, 921–929 (2008).

3. Wong, A. Y. S. *et al.* The Variation of Psychopharmacological Prescription Rates for People With Autism Spectrum Disorder (ASD) in 30 Countries. *Autism Res.* (2014). doi:10.1002/aur.1391

4. Loth, E. *et al.* The EU-AIMS Longitudinal European Autism Project (LEAP): design and methodologies to identify and validate stratification biomarkers for autism spectrum disorders. *Mol. Autism* **8**, 24 (2017).

5. Rasmussen, C. E. & Williams, C. K. I. Model Selection and Adaptation of Hyperparameters. *Gaussian Process. Mach. Learn. (Adaptive Comput. Mach. Learn. Ser.* 105–128 (2006). doi:10.1142/S0129065704001899

6. Zabihi, M. *et al.* Dissecting the Heterogeneous Cortical Anatomy of Autism Spectrum Disorder Using Normative Models. *Biol. Psychiatry Cogn. Neurosci. Neuroimaging* **4**, 567–578 (2019).

7. Marquand, A. F., Rezek, I., Buitelaar, J. & Beckmann, C. F. Understanding Heterogeneity in Clinical Cohorts Using Normative Models: Beyond Case-Control Studies. *Biol. Psychiatry* **80**, 552–561 (2016).

8. Dale, A. M., Fischl, B. & Sereno, M. I. Cortical surface-based analysis: I. Segmentation and surface reconstruction. *Neuroimage* (1999). doi:10.1006/nimg.1998.0395

9. Rosen, A. F. G. *et al.* Quantitative assessment of structural image quality. *Neuroimage* (2018). doi:10.1016/j.neuroimage.2017.12.059

10. Pedregosa, F. *et al.* *Scikit-learn: Machine Learning in Python Gaël Varoquaux Bertrand Thirion Vincent Dubourg Alexandre Passos PEDREGOSA, VAROQUAUX, GRAMFORT ET AL. Matthieu Perrot*. *Journal of Machine Learning Research* **12**, (2011).

11. Ng, A. Y. & Jordan, M. I. *On Spectral Clustering: Analysis and an algorithm*.

12. Von Luxburg, U. A tutorial on spectral clustering. *Stat. Comput.* **17**, 395–416 (2007).

13. Sidorov, G., Gelbukh, A., Gómez-Adorno, H. & Pinto, D. Soft similarity and soft cosine measure: Similarity of features in vector space model. *Comput. y Sist.* (2014). doi:10.13053/CyS-18-3-2043

14. Dinga, R. *et al.* Evaluating the evidence for biotypes of depression: Methodological replication and extension of Drysdale et al. (2017). *NeuroImage Clin.* **22**, (2019).

15. Rutter, M., LeCouteur, A. & Lord, C. Autism Diagnostic Interview - Revised (ADI-R). *Statew. Agric. L. Use Baseline 2015* **1**, (2015).

16. Lord, C. *et al.* The Autism Diagnostic Observation Schedule-Generic: A standard measure of social and communication deficits associated with the spectrum of autism. *J. Autism Dev. Disord.* (2000). doi:10.1023/A:1005592401947

17. Kraha, A., Turner, H., Nimon, K., Zientek, L. R. & Henson, R. K. Tools to support interpreting multiple regression in the face of multicollinearity. *Front. Psychol.* **3**, (2012).

18. Haufe, S. *et al.* On the interpretation of weight vectors of linear models in multivariate neuroimaging. *Neuroimage* **87**, 96–110 (2014).

19. Desikan, R. S. *et al.* An automated labeling system for subdividing the human cerebral cortex on MRI scans into gyral based regions of interest. *Neuroimage* **31**, 968–980 (2006).

20. Constantino, J. N. & Gruber, C. P. *Social responsiveness scale: SRS-2*. (Western Psychological Services Torrance, CA, 2012).

21. Bodfish, J. W., Symons, F. J., Parker, D. E. & Lewis, M. H. Varieties of repetitive behavior in autism: Comparisons to mental retardation. *J. Autism Dev. Disord.* **30**, 237–243 (2000).

22. Dunn, W. *Sensory profile*. (Psychological Corporation, 1999).

23. Das, S. *et al.* Next-generation genotype imputation service and methods. *Nat. Genet.* **48**, 1284–1287 (2016).

24. McInnes, L., Healy, J. & Melville, J. UMAP: Uniform Manifold Approximation and Projection for Dimension Reduction. (2018).

25. Campello, R. J. G. B., Moulavi, D. & Sander, J. Density-based clustering based on hierarchical density estimates. in *Lecture Notes in Computer Science (including subseries Lecture Notes in Artificial Intelligence and Lecture Notes in Bioinformatics)* **7819 LNAI**, 160–172 (Springer, Berlin, Heidelberg, 2013).

26. Gazal, S. *et al.* Inbreeding coefficient estimation with dense SNP data: Comparison of strategies and application to HapMap III. *Hum. Hered.* **77**, 49–62 (2014).

27. Euesden, J., Lewis, C. M. & O’Reilly, P. F. PRSice: Polygenic Risk Score software. *Bioinformatics* **31**, 1466–8 (2015).

28. Demontis, D. *et al.* Discovery of the first genome-wide significant risk loci for attention deficit/hyperactivity disorder. *Nat. Genet.* **51**, 63–75 (2019).

29. Grove, J. *et al.* Identification of common genetic risk variants for autism spectrum disorder. *Nat. Genet.* **51**, 431–444 (2019).

30. Anney, R. J. L. *et al.* Genetic determinants of common epilepsies: A meta-analysis of genome-wide association studies. *Lancet Neurol.* **13**, 893–903 (2014).

31. Savage, J. E. *et al.* Genome-wide association meta-analysis in 269,867 individuals identifies new genetic and functional links to intelligence. *Nat. Genet.* **50**, 912–919 (2018).

32. Nagel, M. *et al.* Meta-analysis of genome-wide association studies for neuroticism in 449,484 individuals identifies novel genetic loci and pathways. *Nat. Genet.* **50**, 920–927 (2018).

33. Ripke, S. *et al.* Biological insights from 108 schizophrenia-associated genetic loci. *Nature* **511**, 421–427 (2014).

34. Schork, A. J. *et al.* A genome-wide association study of shared risk across psychiatric disorders implicates gene regulation during fetal neurodevelopment. *Nat. Neurosci.* **22**, 353–361 (2019).

35. Hanley, J. A. & McNeil, B. J. *The Meaning and Use of the Area under a Receiver Operating Characteristic (ROC) Curve1*.
